# Drivers and patterns of antibiotic use in small to medium-sized chicken farms in selected districts of Nepal

**DOI:** 10.3389/fvets.2025.1570822

**Published:** 2025-08-06

**Authors:** Jeevan Adhikari, Sharada Thapaliya, Reshmi Munakarmi, Pawan Acharya, Hom Bahadur Basnet, Narayan Paudyal

**Affiliations:** ^1^Department of Veterinary Surgery and Pharmacology, Faculty of Animal Science, Veterinary and Fisheries, Agriculture and Forestry University, Rampur, Nepal; ^2^Department of Veterinary Surgery and Pharmacology, Faculty of Animal Science, Veterinary and Fisheries, Agriculture and Forestry University, Rampur, Nepal; ^3^National Animal Health Research Centre, Nepal Agricultural Research Council, Khumaltar, Nepal; ^4^Department of Veterinary Pathology and Clinics, Faculty of Animal Science, Veterinary and Fisheries, Agriculture and Forestry University, Rampur, Nepal; ^5^Department of Veterinary Microbiology and Parasitology, Faculty of Animal Science, Veterinary and Fisheries, Agriculture and Forestry University, Rampur, Nepal

**Keywords:** drivers and patterns, antibiotics usage, layers, chicken, Nepal

## Abstract

The rise in commercial poultry production in Nepal has led to inappropriate and haphazard use of antibiotics, contributing to antimicrobial resistance (AMR). This study aimed to identify the drivers influencing antibiotic use among small to medium-scale layer chicken farms in selected districts. A cross-sectional questionnaire survey was conducted among 180-layer poultry farmers in Makawanpur (*n* = 45), Chitwan (*n* = 30), Dang (*n* = 46), and Pokhara (*n* = 59). Data were analyzed using descriptive statistics and multivariable logistic regression analysis. Among the 346 responses, antibiotics were used for treatment (50.6%), prophylaxis (32.7%), growth promotion (4.6%), improving egg laying (2.6%), and viral infections (9.5%). Antibiotics were administered during brooding (33.8%), debeaking and vaccination (30%), disease epidemics (23.8%), transportation (7.5%) and shifting of houses (5%). Only 39.1% of decisions regarding antibiotic usage were based on laboratory reports; others relied on professional experience (25.8%), necropsy findings (23.4%), or telemedicine consultations (11.7%). Correct dose measurement using graduated dispensers was reported in only 27.2% of responses. Fluoroquinolones were the most used (22.2%), followed by tetracyclines (19%) and macrolides (17.6%). The multivariable logistic regression analysis revealed that poor biosecurity scores and higher stress levels in birds significantly increased antibiotic use at the farm level. Our findings indicate that inadequate knowledge among poultry producers leads to inappropriate antibiotic use beyond therapeutic purposes. Poor biosecurity practices and stressors are major factors associated with increased antibiotic usage. Creating antimicrobial resistance awareness and promoting responsible use of antibiotics among farmers is essential. An integrated national AMR and food safety policy, including farmer education and strict guidelines, would be beneficial.

## 1 Introduction

Poultry farming has undergone a massive transformation in Nepal. The poultry sector, estimated to contribute 3.5% to the national gross domestic product (1) is rapidly emerging as an organized industry and positively impacts humans by ensuring the supply of affordable and sustainable protein sources (2). Nepal now produces 1.61 billion eggs and 548,000 tons of meat annually and has been declared self-sufficient in chicken eggs and broiler meat by the government (3).

The increasing commercialization of the poultry sector has led to an increase in the demand for antibiotics (4). Antibiotics are used in poultry feed at sub-therapeutic levels for improved efficiency and growth promotion, which positively contributes to bacterial resistance by continued selection pressure (5). Consumption of veterinary antibiotics increases mainly due to rampant use without a veterinarian’s prescription and ease of access, which lures excess use through retailers (6). In Nepal, macrolides, cephalosporins, polymyxins, and quinolones are among the commonly used antibiotics in poultry farms for therapeutic purposes or prophylaxis (7). Antibiotics such as chlortetracycline, tylosin tartrate, bacitracin methylene disalicylate, neomycin, lincomycin, and doxycycline were also frequently used in poultry feed as additives or growth promoters (8), but now such in-feed usage has been deemed illegal by the law. The high prevalence of infectious diseases, lack of awareness of good management practices (GMP), lack of observance of drug withdrawal periods, and inadequate diagnostic facilities have contributed to increasing antibiotic resistance in the poultry sector in Nepal (9). A study showed that a large proportion of Nepalese poultry are infected with pathogenic bacteria like Salmonella, *E. coli*, and *Enterococcus,* with antibiotic resistance ranging between 13–17% for *Enterococcus*, 37–44% for *E. coli,* and 45–46% for Salmonella (10).

The data on drivers and patterns of antibiotic use in small to medium-scale layers poultry farms in Nepal is limited, as prior studies primarily focused on broiler production systems (11) or aggregated layer-specific results with broilers (7). In this context, this study was conducted to understand the usage patterns of antibiotics in layer poultry production and identify of knowledge, practices, and attitudes of these farmers regarding antibiotics. The findings of this study will be valuable for policy development and for facilitating interventions for minimizing the threat of antimicrobial resistance (AMR) in animal production, in line with the theme envisioned in the National Action Plan against Antimicrobial Resistance in Nepal (NAP-AMR).

## 2 Materials and methods

### 2.1 Study design and area

**Figure 1:**
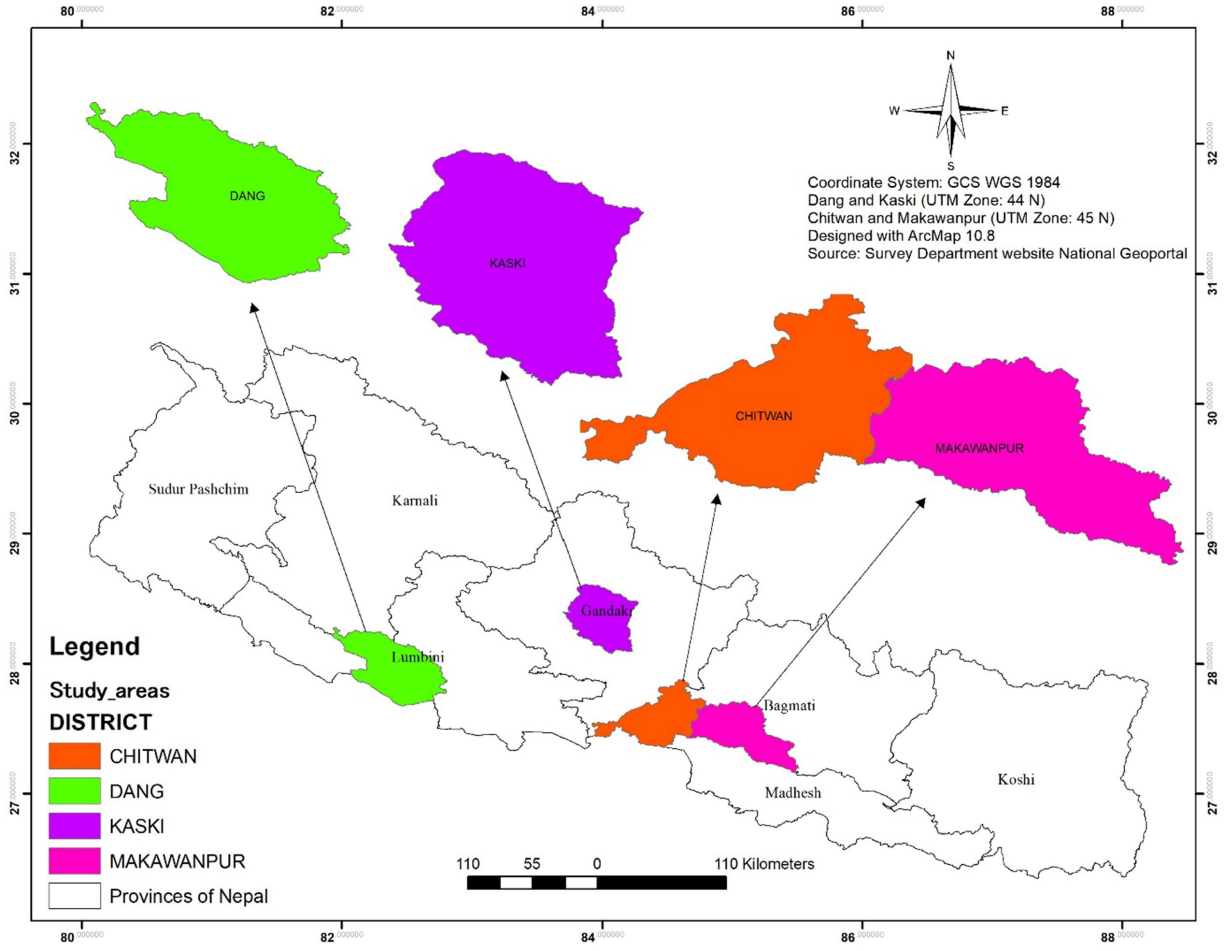
Study area shown within the map of Nepal.

A cross-sectional survey was conducted from March 2020 to March 2023 in four purposively selected districts (Pokhara, Chitwan, Makawanpur, and Dang) in Nepal (Figure 1). The study areas were selected based on the high number of layers in poultry farms in these regions. From a list of all the registered layer farmers provided by the District Poultry Entrepreneurs Forum, we purposively selected small to medium-scale farms (100–5,000 birds) that were in operation for more than 1 year to participate in this study. Large-scale commercial farms and mixed poultry farming were excluded from our study. The study sample size was calculated using Slovin’s formula (1960) as: *n* = (N/1 + Ne^2^), where, *n* = population size (301); *e* = margin of error (0.05 for a 95% confidence level).

By using this formula, the required sample size was calculated to be 172. Altogether, 180 farmers were surveyed; 45 in Makawanpur, 30 in Chitwan, 46 in Dang, and 59 in Pokhara. Pre-testing of the questionnaire was done with 10 farmers not involved in the actual survey to ensure clarity and check if the necessary information was adequately captured during the survey. The questionnaire was modified by incorporating the inputs received and changes required. The pre-test responses are not included in the final dataset.

The study did not include any invasive animal experiments; thus, no ethical clearance was necessary for the farm sampling. Similarly, there were no invasive experiments on human subjects (farmers) as well. Informed oral consent was obtained from each farmer before the questionnaire survey and sample collection. Farmers themselves decided whether or not to participate in this study. Only those farmers willing to participate were included in the study.

### 2.2 Data collection and analysis

The questionnaire format was designed in EpiCollect5, a free mobile and web-based application for data collection (12). The study team conducted a debriefing session with the field investigators/enumerators before their deployments to collect data on the knowledge, attitudes, and practices regarding antibiotic use (ABU) through personal interviews. Survey data were collected on handheld mobile devices using the EpiCollect5 application. The questionnaire was split into multiple sections, incorporating relevant questions on demographics, knowledge, practices, attitudes, feeding practices, antibiotic usage, biosecurity measures, and farm characteristics. The farmer’s interview was complemented with visual assessments of farm management practices and stressors. Litter quality was evaluated by pressing a handful of litter in the hand: damp litter adhered tightly, while dry litter failed to adhere. Farm sanitation was assessed based on the presence of functional footbaths, general cleanliness around the farm, and the use of disinfectant. Stocking density was calculated by the number of birds per square foot. Farm orientation/construction and dirty feeders/drinkers, tall thick vegetation in the farm as observed, and poor ventilation, evaluated by ventilation areas within the shed, were also recorded during the survey. The completed entries were downloaded from the EpiCollect5 servers in MS Excel format, curated, and reorganized for further analysis. Descriptive analysis was carried out in GraphPad Prism v8 for the responses obtained from poultry farmers regarding antibiotic usage patterns. Multiple responses were expressed as frequencies and percentages. Multivariable logistic regression analyses were performed in SPSS v28 for drivers/factors that influence the use of antibiotics at the farm level. The goodness of fit was determined by using the likelihood ratio chi-square test and McFadden’s Pseudo R^2^. The model showed a good fit, with LR X^2^ = 31.95, *p* = 0.0041 and a Pseudo R^2^ value of 0.23, indicating this model is appropriate for multivariable logistic regression. A *p*-value<0.05 was considered significant. We used antibiotic use as a dependent variable and other farm factors as independent variables. The values obtained for the odds ratio (OR), standard error, *z*-statistic, *p*-value, and 95% confidence interval provide the relationship between these variables. The log odds of the dependent variable are associated with a one-unit increase in the independent variable, whereas the other variables remain constant. An OR greater than 1suggests increased odds while less than 1 suggests decreased odds. The standard error estimates the coefficient variability. A smaller standard error indicates a more precise estimate. The *z*-statistic, calculated as the odds ratio divided by its standard error, is used to test whether the coefficient is significantly different from zero. The *p*-value<0.05 is considered a significant association. The confidence interval provides a range within which the true coefficient is likely to lie with a 95% confidence. If the interval does not include zero, the association is statistically significant.

## 3 Results

The results are segregated into several broad thematic groups to facilitate the analysis and interpretation.

### 3.1 Farm demographics

Our survey of 180 farmers regarding their awareness of antibiotic resistance revealed several critical insights. The majority were male, 83.33% (*n* = 150), with females comprising only 16.67% (*n* = 30). The age distribution indicated that 23.89% (*n* = 43) were between 21 and 30 years old, 38.33% (*n* = 69) were 31–40 years old, 26.11% (*n* = 47) were aged 41–50, 10% (*n* = 18) were 51–60, and a small fraction, 1.67% (*n* = 3), were above 60 years. Educational attainment among the farmers showed that 50.56% (*n* = 91) had primary education, 36.11% (*n* = 65) had high school education, and 13.33% (*n* = 24) attained tertiary education. Occupational roles were largely skewed toward production only, with 92.78% (*n* = 167) involved in this vocation. In addition to the production, 3.89% (*n* = 7) were involved in slaughter slabs, 1.67% (*n* = 3) in veterinary pharmacy, and 1.67% (*n* = 3) in feed trade and supply.

Training on poultry health, biosecurity, production, and marketing was received by 46.11% (*n* = 83), whereas slightly more than half of the participants, 53.89% (*n* = 97), reported not having received training of any kind. While 60.56% (*n* = 109) could explain the basic functions of antibiotics, a substantial proportion of 39.44% (*n* = 71) thought any medicine was an antibiotic and antibiotics were just one other kind of medicine. They could not distinguish between critical medicines (e.g., antibiotics) and supplements (e.g., liver tonics, toxin binders, or vitamin supplements). More strikingly, only 21.67% (*n* = 39) could explain antimicrobial resistance, whereas 78.33% (*n* = 141) were unaware of this critical issue.

Regarding the use of probiotics in poultry, only 32.78% (*n* = 59) were aware, while 67.22% (*n* = 121) could not distinguish it from other medicines that they regularly use in their birds.

For the dosage of the antibiotic, 57.78% (*n* = 104) were able to explain it, and 42.22% (*n* = 76) were unable to do so. Knowledge about vaccines was relatively higher, with 76.11% (*n* = 137) understanding their use, yet 23.89% (*n* = 43) lacked this knowledge. Among the farmers, 56.11% (*n* = 101) regularly vaccinated their flocks. The details of these demographic variables are summarized in Table 1.

**Table 1:** Demographic features of farmers and farm practices (*N* = 180).

Variables	Features	Frequency (N)	Percent (%)
Gender	Male	150	83.33
	Female	30	16.67
Age (Years)	21–30	43	23.89
	31–40	69	38.33
	41–50	47	26.11
	51–60	18	10.00
	Above 60	3	1.67
Education	Primary (Up to 5)	91	50.56
	High School (Up to +2)	65	36.11
	Tertiary (Bachelors or Above)	24	13.33
Vocation	Only production	167	92.78
	Slaughter slabs	7	3.89
	Vet. pharmacy	3	1.67
	Feed supplier	3	1.67
Training in poultry health, biosecurity, and production	Yes	83	46.11
	No	97	53.89
Able to explain the effects of antibiotics	Yes	109	60.56
	No	71	39.44
Able to explain antimicrobial resistance	Yes	39	21.67
	No	141	78.33
Able to explain the use of probiotics in poultry	Yes	59	32.78
	No	121	67.22
Able to explain the dosage of antibiotics used	Yes	104	57.78
	No	76	42.22
Able to explain vaccines’ use	Yes	137	76.11
	No	43	23.89
Vaccination	Yes	101	56.11
	No	79	43.88

### 3.2 Patterns of antibiotics use

**Figure 2:**
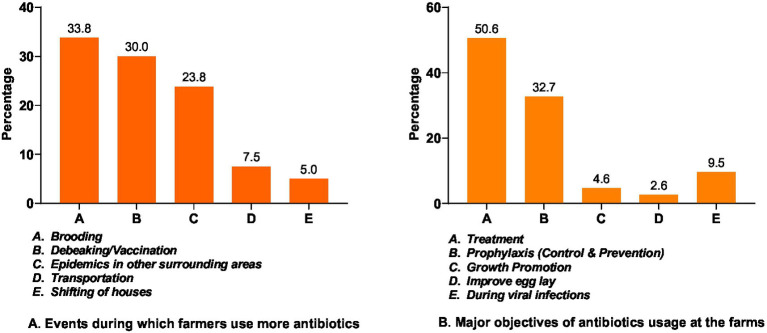
Events and objectives of antibiotics usage in the layer farms.

Farmers used antibiotics for multiple events within a single flock (Figure 2A). Due to multiple responses (i.e., more than one answer per farmer), 240 responses were collected from 180 farmers. Antibiotics were most frequently used during brooding, 33.8% (*n* = 81); followed by debeaking and vaccination, 30.0% (*n* = 72); during epidemics, 23.8% (*n* = 57); before or during transportation, 7.5% (*n* = 18), and during shifting of houses, 5% (*n* = 12).

From 346 responses obtained from 180 farmers regarding the objectives of antibiotic use in a single batch, 50.6% (*n* = 175) reported using antibiotics for treatment, while 32.7% (*n* = 113) used them as prophylaxis for disease prevention purposes. Additionally, antibiotics were used as growth promoters by 4.6% farmers (*n* = 16), for improving egg lay in 2.6% (*n* = 9), and during viral infections in 9.5% (*n* = 33) (Figure 2B).

**Figure 3:**
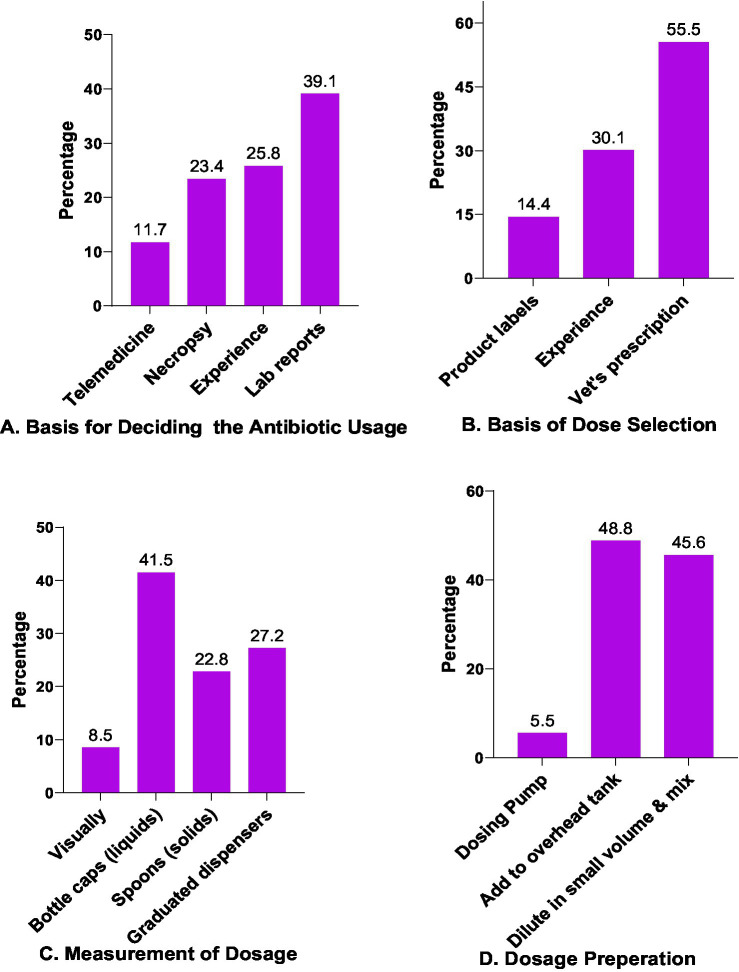
Antibiotic selection, dosing, measurement, and preparation.

In this study involving 180 farmers, a total of 248 responses regarding decision-making processes for antibiotic usage was collected. Only 39.1% (*n* = 97) of the decisions were based on laboratory reports for antibiotic treatments. In contrast, 25.8% (*n* = 64) depended on their own professional experience, while 23.4% (*n* = 58) based their decisions on necropsy findings. Some 11.7% (*n* = 29) used telemedicine consultations before antibiotic use (Figure 3A).

In our survey examining the basis of dose selection of antibiotics (total responses 229 obtained for 180 farmers), the majority of responses, 55.5% (*n* = 127), were reported following a veterinarian’s prescriptions. In contrast, 30.1% (*n* = 69) relied on their own experience or previous prescriptions for dose estimation. Only a small fraction, 14.4% (*n* = 33), determined the dose based on product labels (Figure 3B).

The measurement of the required dose/volume was also studied. A total of 316 responses were obtained from 180 farmers. Among them, 27.20% (*n* = 86) reported correct measurement, i.e., through graduated dispensers. The remaining responses relied on incorrect methods, viz. 8.5% (*n* = 27) measured doses visually, 22.8% (*n* = 72) used spoons, and 42.0% (*n* = 131) used bottle caps for measurement (Figure 3C).

We also evaluated methods for the preparation and dispensing of antibiotics, collecting 217 responses in total. Among these, 48.8% (*n* = 109) reported adding antibiotics directly to the overhead tank. A similar proportion, 45.6% (*n* = 99), preferred diluting antibiotics in small volumes before mixing them into the overhead tank. Only a small proportion of responses, 5.5% (*n* = 12), used dosing pumps for antibiotic mixing (Figure 3D).

### 3.3 Duration of treatment

**Figure 4:**
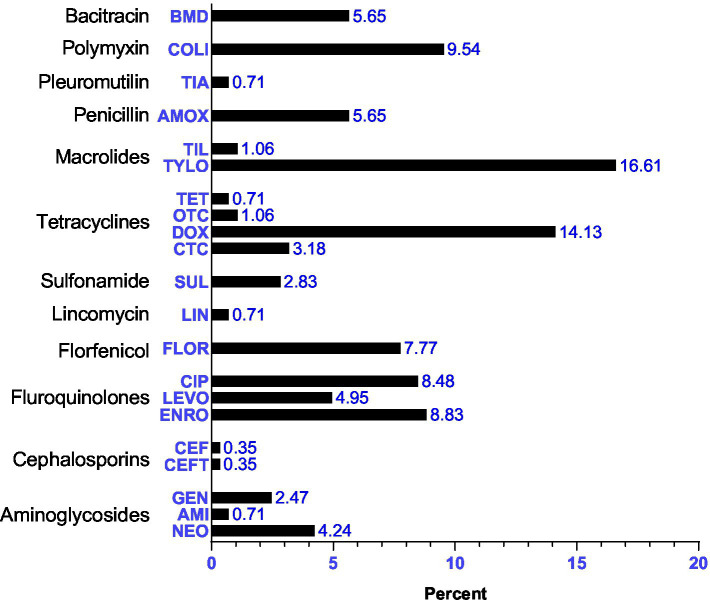
Commonly used antibiotics. The names of the antibiotic molecules on the left Y-axis are abbreviated as: bacitracin methylene disalisylate (BMD); colistin sulfate (COLI); tiamulin (TIA); amoxycillin (AMOX); tilmicosin (TIL); tylosin (TYLO); tetracycline (TET); oxytetracycline (OTC); doxycycline (DOX); chlortetracycline (CTC); sufamethoxazole (SUL); lincomycin (LIN); florfenicol (FLOR); ciprofloxacin (CIP); levofloxacin (LEVO), enrofloxacin (ENRO); cephalexin (CEF); ceftiofur (CEFT); gentamicin (GEN); amikacin (AMI) and neomycin (NEO).

Our study on the duration of antibiotic treatment patterns collected 180 responses. It revealed that the majority 73.9% (*n* = 133) followed a 3–5 days’ treatment course. A small proportion of 21.1% (*n* = 38) extended therapy duration up to 7 days while only 2.2% (*n* = 4) treated for less than 3 days. Notably, a limited 2.85% (*n* = 5) continued treatment for 10 days or more. Following the completion of the antibiotic treatment, our study identified significant variation in the management of the leftover antibiotics. A majority 67.2% (*n* = 121), kept the leftover antibiotics for future use in the next flocks or for treating similar symptoms in the future. Conversely, the remaining, 32.8% (*n* = 44) disposed of the remaining antibiotics, mostly into municipal waste or buried them in the ground (Figure 4).

### 3.4 Commonly used antibiotics

**Figure 5:**
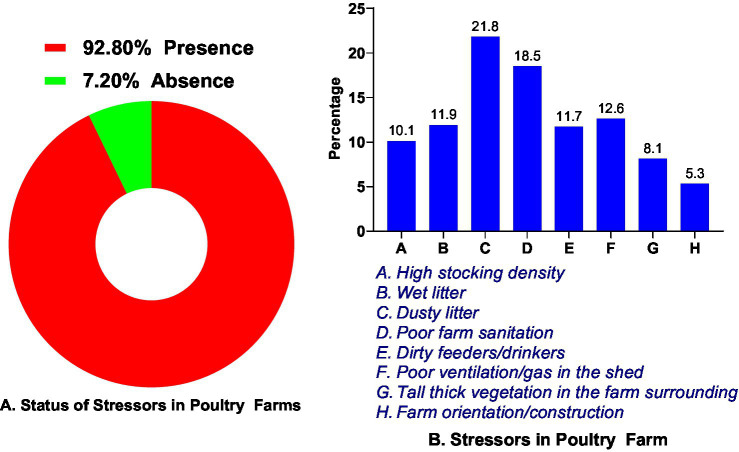
Stressors, as observed by the interviewer during the face-to-face farmers survey in the layers’ poultry farm.

Fluoroquinolones were the most commonly used group of antibiotics (22.26%) followed by tetracyclines (19.08%) and macrolides (17.7%) as illustrated in Figure 5.

### 3.5 Factors contributing to antibiotics usage

During our field survey, we complemented farmer interviews with visual assessments of farm management practices and stressors. Based on these observations, 92.8% showed the presence of at least one stressor (Figure 5A), with dusty litter being the most common stressor, 21.8%, followed by poor farm sanitation, 18.5%. Farm orientation or construction represented the least prominent stressor, accounting for only 5.3% (Figure 5B).

### 3.6 Multivariable logistic regression analyses

Our study highlights the major factors driving the use of antibiotic among Nepalese farmers. Multivariable analyses revealed that poor biosecurity scores (OR = 1.648, *p* = 0.0476) and higher stress levels (OR = 1.707, *p* = 0.029) significantly influenced antibiotic use at the farm level (Table 2).

**Table 2:** Multivariable logistic regression of antibiotic uses model (*N* = 180).

Antibiotics use	Odds ratio	Std. Err.	*z*	*p* > |*z*|	95% confidence interval	Sig
Vaccination status	1.017	0.55	0.03	0.975	0.352	2.937
Poor biosecurity Score	1.684	0.441	1.99	0.047	1.008	2.814**
Stressor level score	1.707	0.419	2.18	0.029	1.055	2.761**
Gender	0.446	0.392	−0.92	0.358	0.08	2.495
Age	0.85	0.222	−0.62	0.533	0.509	1.418
Education	1.186	0.499	0.40	0.686	0.52	2.706
Occupation	0.769	0.38	−0.53	0.595	0.292	2.027
Training	1.746	1.081	0.90	0.368	0.519	5.874
Able to define antibiotics	1.03	0.774	0.04	0.969	0.236	4.491
Able to explain the effects of antibiotics	2.887	1.864	1.64	0.101	0.814	10.237
Able to explain antimicrobial resistance	2.423	2.455	0.87	0.383	0.332	17.658
Able to explain the use of probiotics	0.75	0.527	−0.41	0.682	0.189	2.977
Able to explain the dosage of antibiotics	1.006	0.609	0.01	0.993	0.307	3.293
Able to explain the vaccines’ use	0.578	0.454	−0.70	0.485	0.124	2.691
Constant	1.151	1.661	0.10	0.922	0.068	19.469
Mean dependent var.	0.872		SD dependent var.		0.335	
Pseudo r-squared	0.232		Number of obs.		180	
Chi-square	31.947		Prob > chi2		0.004	
Akaike crit. (AIC)	135.623		Bayesian crit. (BIC)		183.518	

*** *p* < 0.01, ** *p* < 0.05, * *p* < 0.1.

## 4 Discussion

This study gives valuable insights into the complex interplay of farmer behavior, farm practices, and different factors that drive antibiotic use in small and medium farms in Nepal. The farmers in our study accepted the multi-functional use of antibiotics beyond therapeutic needs. Similar multifunctional usage patterns have been observed earlier, where 78% of farms were used for therapeutic purposes and 22% for prophylaxis purposes (11). During the brooding of chicks, antibiotics are commonly used to reduce chick mortality (13). Although only a relatively small percentage of respondents used antibiotics for growth promotion (4.6%), both human and animal health organizations have denounced all growth promotion use (14).

Despite having access to veterinary laboratories, most farmers in Chitwan did not utilize such services due to the long turn around time for receiving results (15). In the absence of proper diagnosis and testing of the susceptibility of various antibiotic molecules, farmers tend to overuse the antibiotics. Educating farmers on the importance of incorporating laboratory diagnostics into their routine practices is essential for promoting responsible antibiotic use, safeguarding animal health, and minimizing the risks of AMR spreading through the food chain (16). Veterinarians are trusted advisors to farmers and are well-positioned to promote changes in farmer behavior by informing and advising about appropriate antibiotics usage (17).

In our study, although a significant proportion of farmers relied on veterinary prescriptions for dose selection, most farmers measured the calculated doses using bottle caps or spoons. Many farmers directly added the required dosages to the overhead tank, highlighting prevalent inaccuracies in dosing practices among farmers. The majority of farmers had only a primary and high school education level, so farmers’ reliance on traditional practices, which they perceive as reliable, along with limited knowledge about appropriate dosing, often leads to inaccurate dosing practices (18). Antibiotic use, either appropriate or inappropriate, is influenced by demographics and antibiotic knowledge (19). Lack of education, lack of professional farm training, and not getting advice from the veterinary doctors have been reported to be the common reasons behind antibiotic misuse in poultry farms (18).

Veterinarians prescribe the treatment duration based on disease severity, while farmers use it based on product labels or clinical symptoms, so this type of antibiotic use pattern might not completely cure the disease. Cost is another factor contributing to the use of antibiotics. The availability of antibiotics in poultry farming can lead to their excessive use, not only for treating illness but also as a preventive measure or growth promoter, which is a common practice. When antibiotics are stored for future use without proper knowledge or veterinary guidance, they are often used inappropriately, such as administering incorrect dosages, using them for the wrong conditions, or treating healthy birds as a preventive measure (20). Educating farmers about the dangers of storing and misuse of antibiotics, along with enforcing stricter regulations on antibiotic disposal and usage, ensures good poultry farming practices (21). For reducing the unwanted use of antibiotics, the skill of a good facilitator who can bring farmers’ knowledge and experience together with veterinary expertise to empower farmers would impart long-lasting changes to reduce such reliance (17).

One-third of the respondents discarded the leftover antibiotics, with 64.4% disposing-them-off along with household garbage. The use of leftover antibiotics is a common issue in developing countries due to multiple factors such as financial constraints and access to veterinary physicians (18). This practice can lead to environmental contamination and contribute to the development of antibiotic resistance. The indiscriminate release of antibiotics into the environment undermines their efficacy and exacerbates resistance, as harmless microbes can mutate into deadly, resistant pathogens (22).

In the current study, a total of 21 types of antibiotics belonging to 12 different classes were used for both therapeutic and non-therapeutic purposes. Out of them, fluoroquinolones, tetracyclines, macrolides, and polymyxins were the most frequently used classes of antibiotics. The high usage of molecules of fluoroquinolones and macrolides classes, listed as the highest priority critically important antimicrobials (HPCIAs) in human medicine (23) is worrisome. Colistin is considered a last-resort antibiotic for the treatment of infections caused by *Enterobacteriaceae* and *Pseudomonas aeruginosa*. Similarly, antibiotics such as tylosin and ciprofloxacin are used for the treatment of zoonotic diseases caused by pathogens like *Campylobacter* spp., *E. coli*, *Salmonella*, and *Shigella*. Therefore, these antibiotics should not be administered to food-producing animals, including chickens, in the absence of clear clinical signs of disease (14). Although the Government of Nepal has banned the use of colistin in animals since 2018, our data showed that it remains a high-priority antibiotic in poultry production, which could be the result of a weak regulatory mechanism. A study conducted in Chitwan reported that colistin was the first-choice drug in poultry farms (15). Antibiotics such as doxycycline (22%), colistin (15.67%), levofloxacin (14.47%), enrofloxacin (11.84%), and tylosin (10.53%) were also frequently used in poultry farms in the Kaski district (24). This practice can contribute to the development of antibiotic resistance, which is a growing concern in both animal and human health. The correlation between fluoroquinolone usage and resistance was very strong in food-producing animals (25). Tetracyclines are the most affordable broad-spectrum antibiotics, widely used due to their low cost and wide availability (26).

Poor biosecurity practices increase the exposure of poultry to pathogens, leading to more frequent and severe disease outbreaks. In response, farmers may use antibiotics more frequently, sometimes prophylactically, to manage these diseases. This can lead to inappropriate antibiotic use, including overuse and misuse, which contributes to the development of AMR. The appropriate usage of antibiotics is closely associated with biosecurity measures (27). Proper biosecurity management significantly reduces disease in the flocks (28).

In this study, managing stressors such as high stocking densities, wet and dusty litter, poor farm sanitation, insufficient and dirty feeders and drinkers, inadequate ventilation in sheds, and tall, thick vegetation present in farms through improved farm management practices could be a key strategy for reducing antibiotic use. Earlier studies have suggested that antibiotics are used in poultry not only for therapeutic purposes but also to mitigate environmental stresses (29).

The vaccination status did not have a statistically significant impact on antibiotic use in our study. These findings seem contradictory, as vaccinations are generally expected to reduce the need for antibiotics by preventing diseases (30). This discrepancy might reflect limitations in current vaccination programs, such as issues with vaccine quality and coverage, cold chain failures, improper vaccine administration, lack of booster doses, sudden outbreaks of new disease variants, and farmers’ lack of trust in vaccines. Furthermore, inadequate cold chain maintenance during transportation and the use of imported vaccines that may not protect against local pathogen strains can reduce vaccine effectiveness (31).

Like any research, this study has some limitations that should be taken into consideration. First, data were collected through interviews and observation, which may have led some farmers to provide desirable answers, potentially affecting the accuracy of the findings. Second, the use of the purposive sampling method could introduce bias into the sample. Finally, it is important to note that the farmers included in this study were from urban areas such as Chitwan, Pokhara, Makawanpur, and Dang. Therefore, the patterns and drivers of antibiotic use may not necessarily same throughout the country.

## 5 Conclusion

This study reveals that many poultry farmers in Nepal are using antibiotics in ways that go beyond just treating sick birds, often for prevention, growth promotion, or even during viral infections. Unfortunately, many farmers rely on experience or advice from para-veterinarians rather than laboratory tests to decide when and how to use antibiotics. Poor biosecurity and high stress on the birds are the major reasons for increased antibiotic use, where proper dosing practices are often overlooked. These findings highlight an urgent need to aware the farmers on the responsible and only necessary usage of antibiotics while implementing stricter guidelines and tougher regulatory mechanisms. A well-rounded national policy on AMR and food safety, combined with better awareness and farm management practices, could help protect both animal and public health.

## Data Availability

The raw data supporting the conclusions of this article will be made available by the authors, without undue reservation.
